# Impact of Short Foot Muscle Exercises on Quality of Movement and Flexibility in Amateur Runners

**DOI:** 10.3390/ijerph17186534

**Published:** 2020-09-08

**Authors:** Iwona Sulowska-Daszyk, Anna Mika, Łukasz Oleksy

**Affiliations:** 1 Institute of Clinical Rehabilitation, University of Physical Education in Krakow, 31-571 Krakow, Poland; anna.mika@awf.krakow.pl; 2 Physiotherapy and Sports Centre, Rzeszow University of Technology, 35-959 Rzeszow, Poland; loleksy@oleksy-fizjoterapia.pl; 3 Oleksy Medical & Sports Sciences, 37-100 Łańcut, Poland; 4 Orthopaedic and Rehabilitation Department, Medical University of Warsaw, 02-091 Warsaw, Poland

**Keywords:** plantar foot muscles, Functional Movement Screen, muscle flexibility, myofascial chains, energy transfer

## Abstract

The flexibility and proper functioning of all myofascial chains are crucial for athletes, especially for long-distance runners. Due to the continuity of the myofascial structures, restrictions in one part of the body may cause excessive tension in others. The aim of our study was to evaluate the influence of short foot muscle exercises on muscle flexibility and the quality of movement patterns in amateur runners. Eighty long-distance runners, aged 20–45, were randomly divided into two groups: Group 1 (*n* = 48) and Group 2 (*n* = 32). Participants in Group 1 performed foot exercises daily for six weeks. Subjects in Group 2 were without any intervention. At baseline and after six weeks, the quality of movement patterns with the Functional Movement Screen and muscle flexibility was evaluated. In Group 1, significantly higher Functional Movement Screen values in individual tasks and in the total score were noted after six weeks. The total score increased from 17 to 18 points (Median (Me) ± half of interquartile range (IQR/2) (Standard Error of Measurement - SEM) 17 ± 1.5 (0.23) at baseline and 18 ± 1.5 (0.24) after six weeks) (*p* < 0.01), whereas in Group 2, its level remained at 16 points (Me ± IQR/2 (SEM) 16 ± 1.5 (0.31) at baseline and 16 ± 1.25 (0.31) after six weeks). In Group 1, the significant improvement in muscle flexibility was noted (e.g., results for external rotation muscles: (Mean ± SD (SEM) 60.3 ± 0.4 (1.50) at baseline and 62.4 ± 10.3 (1.49) after six weeks) (*p* = 0.005). In Group 2, significant improvement was observed only for one task in the Active straight leg raise test (*p* = 0.005 and 0.02). During the measurement of external rotation muscles, a significant decrease in flexibility was observed (Mean ± SD (SEM) 60.1 ± 9.0 (1.60) at baseline and 58.0 ± 8.5 (1.51) after six weeks) (*p* = 0.001). Plantar short foot muscle exercises may improve muscle flexibility in the upper parts of the body within myofascial chains and influence the quality of fundamental movement patterns. Such exercises may be beneficial for all physically active people and can be performed as part of overall fitness programmes. Moreover, including such exercises in daily training routines of long-distance runners, as well as by athletes in other sport disciplines is also recommended.

## 1. Introduction

Functional movement patterns require the combination of mobility, stability, strength, coordination, and neuromuscular control [1,2]. Such movement patterns are required for the proper performance of more complex and specific athletic tasks and movement patterns, such as running or throwing [3]. The correct interaction between elements of body chains is also necessary to adequately perform different movement patterns. Any dysfunctions in particular parts have an impact on other elements of the kinematic chain. Abnormalities found in distal segments may influence proximal parts of the body and vice versa. For example, excessive pronation transmitted to the internal rotation of the tibia may cause overloading of the knee joint or may be the cause of other changes in proximal parts of the lower extremity [4]. 

Within the lower extremity, in the kinematic chain, a crucial element is the foot. When walking or running, foot pronation occurs, which is a combination of rearfoot eversion, forefoot abduction, and ankle dorsiflexion [5,6]. One of the factors influencing the range of foot pronation and medial longitudinal arch posture is the strength of the plantar intrinsic foot muscles [7]. Their weakness causes changes in foot posture towards a pronated position [8,9,10]. Excessive pronation or supination may cause dysfunction or overloading of the knee joint and other parts of the kinematic chain [6]. The results of our previous study indicate that exercising these muscles significantly modifies foot posture towards a neutral position and reduces the tendency towards pronation [11]. In some studies, the dependence between functional deficits of the plantar fascia, shortening of hamstrings, and restricted range of motion in the ankle joint are suggested [12,13]. Subjects with plantar fasciitis present tightness of the lower limb posterior muscles and a decreased range of ankle dorsiflexion [14,15]. Tautness of the triceps surae and hamstrings may decrease the range of motion in the ankle. The above results may be explained by the existing relationship between the foot and the upper segments of the body via myofascial chains, which connect adjacent muscular structures within the fascial system. The tension and overloading forces are transferred along them. The plantar intrinsic foot muscles are a part of the superficial back line, which also includes the plantar fascia, Achilles tendon, triceps surae, hamstrings, sacrotuberous ligament, fascia of the sacrolumbar area, erector spinae, and epicranial fascia [16,17]. Fascial restrictions in one part of the body may cause excessive tension in other parts due to fascial continuity [18]. 

The sensorimotor system includes plantar receptors of the foot, ankle joint, and the musculotendinous, which have an influence on sensorimotor control [19,20,21]. It was reported that plantar foot massage or ankle joint mobilisation might improve sensorimotor function in patients with chronic ankle instability [19]. Conversely, other authors have suggested that the restrictions within the ankle ligamental system may have adverse effects on postural control [22].

There are some studies in which the influence of different kinds of therapy within foot muscles on the upper segments of the body was indicated. It was reported that the application of the self-myofascial release technique on the foot plantar surface might improve the flexibility of the hamstrings [23]. On the other hand, in a different study, the authors reported opposite results. They observed that unilateral foot rolling did not increase ankle dorsiflexion or range of motion in the Sit and Reach test [24]. Some authors noted that intrinsic foot muscle exercises might increase foot muscle strength and athletic performance [10,25]. In a previous study, the authors observed improvement in some functional movement patterns after implementing foot exercises [11]. There are also some studies in which their authors have reported the influence of the myofascial release technique performed within the proximal part of the lower extremity, excluding the foot. These researches found therapeutic effects in elements distant from the area where the technique was applied [26,27,28]. 

To achieve a proper running technique, fundamental elements concern mobility and stability of whole-body patterns, which are assessed using the Functional Movement Screen test (FMS), which is a comprehensive test for determining the quality of basic movement patterns, requiring the combination of stability, mobility, strength, and coordination of the involved muscle groups and proper neuromuscular control. For runners, the key elements are mobility of the ankle, hip, and thoracic segment (allowing free movement of trunk rotation during running), as well as stability within the knees, pelvis, and trunk. All these elements are evaluated using the FMS test; therefore, this tool can be used to effectively monitor the quality of mobility and stability in runners, which is directly related to the quality and efficiency of movement during the run [29].

There are some studies in which a concern is put forward, stating that chronic endurance training may induce decreased flexibility [30,31]. An optimal level of this parameter is necessary for health. Higher muscle flexibility can produce a protective role against muscle damage during exercise [32,33].

Long-distance running is one of the most popular sport disciplines wherein athletes are exposed to repetitive loads; thus, improving the quality of fundamental movement patterns should be included in the basic elements of training because it may reduce the risk of injury [5,34]. Our study is the first in which it was verified whether exercising the short foot muscles can enhance flexibility within myofascial chains, and through it, improve the quality of movement patterns. The aim of our study was to evaluate the influence of plantar short foot muscle exercises on myofascial flexibility in the upper parts of the body, and on the quality of fundamental movement patterns in long-distance runners.

## 2. Materials and Methods 

### 2.1. Study Group

The study included 110 runners, of which, 30 participants discontinued the experiment and were not included in further analysis. Finally, 80 long-distance, recreational runners (23 females and 57 males), aged 20–45 years (mean ± SD 32.85 ± 7.22), who regularly run a total distance of 20–100 kilometres (km) per week (mean ± SD 44.56 km ± 18.43 km), participated in our study. The inclusion criteria were as follows: age between 20 and 45 years, a weekly running distance of 20 kilometres or more, regular running training (at least three running session per week, with a minimum training time of 30 min), no deformation of the feet in visual assessment, no acute injury six months prior to enrolment in the trial, consent to participate in the study. The inclusion criteria were created on the basis of data from the International Institute for Race Medicine [35]. In 2018, runners at an age between 20 and 40 years, constitute almost 60% of participants in a marathon and five-kilometre races. 

The exclusion criteria were as follows: age above 45 years or less than 20, lack of consent to participate in the study, a weekly running distance of less than 20 kilometres, irregular running training, visible deformation of the feet under visual assessment, previous history of acute injury six months prior to enrolment in the study, chronic pain, systemic disease (e.g., diabetes, fibromyalgia, hypertension).

Before the study, approval of the Ethical Committee of Regional Medical Chamber had been obtained (No. 40/KBL/OIL/2015). All participants were informed in detail about the research procedures and purpose of the study before they provided their written informed consent. All the procedures complied with the Declaration of Helsinki.

The study participants were randomly divided into two groups: Group 1 (*n* = 48), in which subjects performed the foot exercises daily for six weeks; and Group 2 (*n* = 32), without any intervention. The researchers used simple randomisation by flipping a coin. The researcher was blinded to the subject group allocation. A detailed characterisation of both groups is presented in Table 1.

### 2.2. Procedures

The runners from Group 1 were familiarised with the exercise protocol and received verbal and written instructions. The subjects performed exercises of the plantar short foot muscles daily, lasting 30 min, for six weeks. Once a week, the training was supervised by a physical therapist, who examined the correctness of exercises performance. Moreover, every day, the participants received a reminder about the requirement to perform the exercises, which they confirmed doing. The study protocol included the progression of exercises by increasing the load and level of difficulty every two weeks. The following tools were used: a tennis ball, stability disc, and a band loop. In each exercise, participants paid attention to the proper load on three support points: the heel and the heads of the 1st and the 5th metatarsals. The exercises were performed barefoot. The protocol included exercises activating the plantar short foot muscles [36,37]. 

Before each training session, the participants performed plantar self-myofascial release of each foot for 5 min using a tennis ball. The basic exercise was the Short Foot Exercise, which consisted of two stages: the 1st: the participants were instructed to shorten the foot in the anteroposterior direction by bringing the heads of metatarsal bones towards the heel without toe flexion. Then, balanced loading of the three support points of the foot was performed: the heel and the heads of the 1st and the 5th metatarsals, maintaining shortened position. During the exercises, the feet maintained on the ground and the toes were relaxed. The exercise was repeated 30 times. The progressions included performing this exercise in seated, standing, and half-squat positions.

The Reverse Tandem Gait exercise was performed by walking backward in a straight line with the arms alongside the body and one foot directly behind the other. In the beginning, the metatarsus was loaded with an equal load on the 1st and the 5th metatarsals, and then the heel. The runners took 30 steps.

The Vele’s Forward Lean was based on the maximal forward lean from the standing position. The exercise was performed with the feet shoulder-width apart, with arms alongside the body, keeping heels on the ground and the body in alignment. During this exercise, the short foot position was maintained with the proper loading of the foot support points. The participant maintained this position for 5 s and repeated it 20 times.

The study participants performed exercises with band loops strengthening the muscle-ligament structures and also exercises on a stability disc. On the stability disc, several exercises were performed, such as maintaining a standing position on the stability disc in double-leg standing and in one-leg standing, alternate ascending on the heel and on the front foot, bearing in mind the proper load of heads of the 1st and the 5th metatarsal bones, or performing half-squats maintaining a standing position. The complete exercise protocol lasted approximately 30 min.

In Group 2, there was no intervention during the six weeks. All measurements were performed at baseline and after six weeks. The running training routine was unchanged and constant throughout the duration of the experiment and was monitored by the investigators. 

### 2.3. Research Tools

The data were collected at the research laboratory. To evaluate the quality of the fundamental movement patterns, the Functional Movement Screen (FMS) test was used [1,2]. The FMS test comprised seven tasks:

(1) Deep squat;
(2) Hurdle step;
(3) In-line lunge;
(4) Shoulder mobility;
(5) Active straight leg raise;
(6) Trunk stability push-up;
(7) Rotary stability.

Each task was evaluated on a four-level scale from 0 to 3. The runners performed all the tasks wearing shoes. During testing, each participant was observed from the front, back, and side. In asymmetric tests, both the right and left sides were assessed, and the lower result was considered in the total score. Each task was performed three times. General evaluation criteria were as follows: three points were accorded when the movement pattern was performed correctly without compensation, two points to movement pattern performed with compensation, while one point meant the inability to execute the task. If pain occurred during the performed test, 0 points were given. All measurements were conducted by the same expert rater. The maximum possible score was 21 points. The reliability of the FMS test was high. Reliability for the Interclass Correlation Coefficient (ICC) intra-rater ranged between 0.81 and 0.91 [38].

Assessment of myofascial flexibility was performed according to Chaitow [39] using a centimetre measuring tape. The following muscles were evaluated:

(1) the rectus femoris muscle was tested in prone position with maximal knee joint flexion; the distance between the lateral malleolus and the table was measured;
(2) the iliopsoas muscle was evaluated using the Thomas test; the distance between the midpoint on the patella lateral edge and the table was measured;
(3) the tensor fasciae latae muscle was assessed in side-lying position with maximal extension, external rotation, and adduction of the hip joint; the distance between the lateral malleolus and the table was measured;
(4) the piriformis muscles (external rotation muscles) were tested in the prone position with maximal internal rotation of the hip joints, and the knee flexed to 90°, the distance between the right and left medial malleoli was measured;
(5) the adductor muscles were evaluated in the supine position with maximal hip adduction and knee extension; the distance between the right and left femoral medial epicondyles was measured;
(6) the quadratus lumborum muscle was assessed in standing position; the displacement in hand finger position between standing relaxed position and maximal side trunk flexion was measured.

### 2.4. Statistical Analysis

Statistical analysis was performed using the STATISTICA 12.0 Pl software (Statsoft Polska, Krakow, Polska). The Shapiro–Wilk test was conducted to assess data for normality. To determine the significance of differences regarding myofascial flexibility measurements, the two-way ANOVA was performed with one main factor being between subjects (Group 1 and Group 2), and the other main factor being a repeated measure (time: baseline and six weeks). Post-hoc analysis was performed using the Tukey’s post-hoc test. Wilcoxon’s non-parametric test was used to assess the significance of the differences concerning the variables tested via the FMS test. To investigate the difference between the groups at baseline and after six weeks, the Mann–Whitney U test was applied. The effect size was calculated using Cohen’s r and interpreted as: trivial (<0.1), small (0.1–0.3), medium (0.3–0.5), and large (>0.5) [40]. Differences were considered statistically significant at the level of (*p* < 0.05).

Using the paired t-test for power analysis of exercise, it was determined that at least 30 subjects from each group were required to obtain a power of 0.8 at a two-sided level of 0.05 with the effect size of *d* = 0.8.

## 3. Results

### 3.1. Functional Movement Screen Test

After six weeks of plantar short foot muscles exercises, higher values were noted for the majority of tasks, and in total score of the FMS test in Group 1. There were significant changes with regard to the following tasks: Deep squat, Hurdle step, In-line lunge, Shoulder mobility with left arm at the top, Active straight leg raise, and Rotary stability (Table 2). In Group 2, a significant change was obtained only in one task of the FMS test (Active straight leg raises) (Table 2).

### 3.2. Functional Tests of Major Muscle Groups

In the functional tests of the major muscle groups compared to baseline after six weeks of exercises, significant improvement in myofascial flexibility of all the evaluated muscle groups was observed in Group 1 (Table 3). In Group 2, a significant change was observed within the external rotation muscles (Table 3).

## 4. Discussion

The most novel finding of our study is that plantar short foot muscles exercises may significantly improve proximal muscles myofascial flexibility and the quality of functional movement patterns in long-distance runners. In the functional tests of the major muscle groups among the experimental group, after six weeks of exercises, in the piriformis muscles (external rotation muscles), tensor fasciae latae, adductor muscles, and the quadratus lumborum, greater values were observed, whereas lower values were noted for the iliopsoas and rectus femoris. All these outcomes indicated improvement related to larger muscles flexibility, and a following increase in the range of motion. In contrast, no improvement was detected for the control group, with a significant decrease of flexibility within the external rotation muscles. Moreover, after six weeks of plantar short foot muscles exercises in the experimental group, significantly higher FMS values for individual tasks and the total score were observed, whereas runners in the control group obtained better results only in the Active Straight Leg Raise (ASLR) task. Higher values obtained in the FMS test indicated improvement in fundamental movement patterns. These effects may be significant in athletic training and will be discussed in further detail below. In the literature available on this subject, there are very few studies in which authors describe the influence of therapy within the foot muscles on the upper segments of the body. However, some researchers undertook this subject of study; nonetheless, their results are only partially related to our current study.

### 4.1. Myofascial System

Previous research is consistent with regard to the existence of the phenomenon of energy transfer through structures of the myofascial system [16,17,18]. In some studies, changes after therapy within the lower limbs, excluding the foot, have been evaluated [26,27,28]. Other researchers focused on foot therapy; however, they assessed the influence of different kinds of myofascial release techniques applied to the foot on proximal parts of the body [23,24]. Nevertheless, none of these studies included therapy within initial parts of the lower limb kinematic chain, based on plantar short foot muscle exercises. Only in our previous studies did we deal with this subject [11,41]. 

Grieve et al. [23] evaluated the effect of a single application of the self-myofascial release technique. This was performed on muscles of the plantar surface of the feet with regard to flexibility of other parts of the superficial back line (hamstrings and lumbar spine) in long-distance runners. They noted a significant improvement in the Sit and Reach tests and suggested that this kind of intervention has clinical benefits for the flexibility of the hamstrings and lumbar spine. Similar to Grieve et al. [23], we noted an improvement in the hamstring flexibility in the Active Straight Leg Raise (ASLR) test. Grabow et al. [24] evaluated the influence of unilateral foot rolling on the ipsilateral and contralateral range of ankle dorsiflexion. They also used the Sit and Reach tests to assess the hamstring and lower back flexibility. However, in contrast to Grieve et al. [23], they did not observe any changes in ankle dorsiflexion. It should be noted that in both of these studies, the investigators assessed the immediate effect of applying a single therapy session using the self-myofascial release technique on the plantar aspect of the feet. 

However, there are more studies of which their authors have reported an influence of the myofascial release technique but performed within the proximal part of the lower extremities. It has been reported that foam rolling applied on the quadriceps femoris muscles significantly increases the range of motion in the knee joints [42]. The same method used for the hamstrings improved the flexibility of these muscles and increased the range of motion in the Sit and Reach test [27,28]. Furthermore, an enhanced range of motion in the ankle after calf muscle foam rolling was reported by Halperin et al. [43]. Hyong and Kang [44] examined the immediate effects of passive hamstring stretching exercises on muscle flexibility and range of motion. The participants of their experimental group underwent hamstring stretching for 30 s, three times with ankle dorsiflexion, whereas the control group received the same therapy protocol without ankle dorsiflexion. The researchers observed that the range of motion in the cervical spine increased only in the experimental group. In conclusion, they indicated connections between muscles within the fascia and force-related interactions between these elements.

All of these studies seem to confirm the existence of connections within the myofascial chains. Our study was the first to assess the effects of long-term foot therapy on the proximal parts of the body within the myofascial system. After six weeks, the improvement of proximal muscles myofascial flexibility was achieved only in the experimental group, whereas in the control group, no changes or decreases in muscle flexibility were observed.

### 4.2. The Plantar Foot as an Element of the Sensorimotor System

The plantar surface of the foot is a part of the sensorimotor system, which consists of plantar receptors of the foot, ankle joint, and musculotendinous receptors [19]. In some studies, it has been described that the stimulation of sensory receptors may improve sensorimotor function [19,20,21,45]. LeClaire et al. [20] have reported immediate effects of plantar foot massage on the improvement of postural control in individuals with chronic ankle instability. In another study, researchers compared the effectiveness of a single bout of three kinds of treatment within the plantar surface of the foot: traditional massage, self-administered with a ball and sensory brush massage. Each of these therapies resulted in comparable static postural-control improvements. The researchers suggested that stimulation of the plantar cutaneous receptors may be the underlying mechanism of postural-control improvement following the plantar massage [45]. McKeon et al. [19] also evaluated the efficacy of the plantar message, triceps surae stretching, and ankle joint mobilisations on the objective and subjective outcome measures regarding sensorimotor dysfunctions and clinical disablement in patients with chronic ankle instability. Both plantar massage and joint mobilisation demonstrated the greatest potential for improvement in sensorimotor function. It was reported that proprioception is crucial for all levels of the central nervous system that provides a sensory component to optimise motor control. Therefore, the motor control system must consider the numerous motions occurring during direct muscle activation and intersegmental dynamics. Thus, proprioception affords the essential position and segmental movement information to the motor control system [46].

### 4.3. Muscle Flexibility and Movement Patterns

The flexibility and proper functioning of all myofascial chains are crucial for athletes, especially long-distance runners. Due to the myofascial structure continuity, restrictions in one part of the body may cause excessive tension in others [17]. Moreover, the overloading forces may also be transferred by the myofascial system, leading to tissue overload, repetitive strain injuries, resulting restrictions in muscles flexibility, and disruptions in functional movement patterns [18]. 

An optimal level of flexibility is necessary for health. According to Gordon and Bloxham [47], flexibility of the muscle, tendons, and ligaments in the back may be associated with range of motion and functional movement. Other researchers suggest that higher trunk flexibility and ankle dorsiflexion values might be associated with a higher ground-force application and lower leg stiffness during running. Moreover, higher muscle flexibility can produce a protective role against muscle damage during exercise [32,33]. In the study performed by Nikolaidis et al. [31], the conclusion was that although flexibility does not relate to marathon performance, it is a component of health-related physical fitness. Researchers suggest that coaches and runners should consider exercises, including stretching in their weekly programme to ensure adequate levels for all components of health-related physical fitness [31].

In our study, we indicated enhancement of muscle flexibility after feet exercises, and a lack of changes in the control group. Therefore, in our trial, beneficial effects of short foot muscles strengthening exercises on myofascial flexibility were revealed, which suggests that these exercises might improve functional movement patterns and, consequently, may potentially reduce the risk of injury in runners. Since the foot is an element of the main myofascial chains—superficial front and back lines, the lateral, and spiral lines [16,17], we suggest that due to myofascial continuity, the exercise performed within the foot may have a beneficial effect on the upper parts of the body. The lack of significant changes in the control group and significant improvement in flexibility of all the evaluated muscle groups (which were parts of several myofascial chains), as well as improvement in functional multidimensional movement pattern quality in the experimental group, might indicate global influence of short foot muscles strengthening exercises. This observation may be supported by a different study [37]. After six weeks of plantar short foot muscles exercises, the authors noted higher values of peak torque, work, and power of the knee joint flexors, which may suggest that strengthening of the short foot muscles may improve the strength of the proximal muscles.

### 4.4. Short Foot Muscles 

The plantar short foot muscles enhance the longitudinal arch and allow to maintain proper relations between the heel and heads of the 1st and the 5th metatarsals. They provide foot stability and flexibility for shock absorption [7]. Dysfunction of the plantar area of the foot may cause problems within the upper parts of the myofascial chains [5]. These functions of the foot are especially significant in long-distance runners, who are liable to long-term and repetitive loadings. The results of our study suggest that short foot muscle strengthening exercises may improve the quality of movement patterns and flexibility within myofascial chains; thus, being potentially beneficial for runners. Better mobility and stability in dynamic movement patterns and better myofascial flexibility may minimise overloading of the foot, and in this way, may potentially further diminish the risk of future injuries in long-distance runners.

In a previous study [11], the authors have noted improvement in some functional movement patterns after exercises within distal parts of the kinematic chain. In this study, runners were divided into two groups: Group 1, in which the participants performed the “Reverse Tandem Gait” and “Vele’s Forward Lean” exercises, and Group 2 with the “Short Foot Exercise”. The authors observed improvement in the deep squat and the active straight leg rise tests among both groups, but the change was only significant in Group 1. The researchers suggested that this difference may result from the greater impact of exercises performed by runners in Group 1 than “Short Foot Exercise” on the musculoskeletal system due to functional and anatomical connections between muscles and the fascia in the superficial back line. Those results were confirmed in our current study, in which we observed higher values of each task and the total score of the FMS test, whereas in the control group, we did not observe any significant changes. The results of our study indicate improvement in the muscle flexibility within myofascial chains and the quality of fundamental movement patterns in long-distance runners.

### 4.5. Study Limitation

There are some limitations to our study that should be addressed. First of all, the number of subjects in the evaluated groups differed. The uneven number of participants in the two groups was caused by the fact that a relatively large number of runners from the control group did not come to the final examination. Therefore, they could not be included in the analysis. Moreover, the total running distance of the participants was between 20 and 100 km per week, which may have influenced the group homogeneity. The FMS test is widely used in mobility and stability assessment, but it is considered as a screening toll, having many weaknesses. These conflicting observations may result from the fact that during the FMS test, body movements are analysed in a general manner; thus some abnormal movement behaviours could have gone undetected. However, the FMS is scored using task-specific criteria, many of them not being biomechanically related to injury mechanisms or also poor FMS performance as well as low composite scores reflecting injury history more than predicting future injury risk.

## 5. Conclusions

Due to the fact that in previous studies, the influence of short foot muscles exercises is assessed only within the foot or lower leg [10,23,24], our study is the first to report the broader impact of these exercises. Based on our results, we suggest that plantar short foot muscle exercises may significantly improve muscle flexibility of upper parts of the body within myofascial chains in long-distance runners. Moreover, these kind of foot exercises may have beneficial effects on the quality of fundamental movement patterns in long-distance runners. Unfortunately, the short foot muscle strengthening exercises are usually ignored by athletes in their training routines. With regard to the results obtained in our study, we suggest that the plantar short foot muscle exercises may be beneficial for all physically active people and can be performed as a part of an overall fitness programme. Moreover, including such exercises in the daily training routine of long-distance runners as well as athletes representing other sport disciplines is also recommended. Short foot muscle strengthening exercises may improve the quality of movement patterns and flexibility within myofascial chains; thus, potentially becoming beneficial for runners. Better mobility and stability of dynamic movement patterns and better myofascial flexibility may minimise overloading of the foot and also potentially diminish the risk of future injuries in long-distance runners.

## Figures and Tables

**Table 1:** Detailed characterisation of groups.

	Group 1 (*n* = 48) Mean ± SD	Group 2 (*n* = 32) Mean ± SD
Age	32.48 ± 6.81	33.41 ± 7.76
Males	31	26
Females	17	6
High [cm]	174.94 ± 8.73	177.69 ± 7.89
Body mass [kg]	69.81 ± 9.68	71.00 ± 10.55
Total distance covered per week [km]	42.19 ± 18.54	48.13 ± 17.67

SD—standard deviation. cm—centimetres. kg—kilograms. km—kilometres.

**Table 2:** The Functional Movement Screen test at baseline and after six weeks of exercising.

Outcome Measure		Group 1 Me ± IQR/2 (SEM)	*p* ^a^	ES ^a^	Group 2 Me ± IQR/2 (SEM)	*p* ^a^	ES ^a^	*p* ^b^	ES ^b^
Deep Squat	B	2 ± 0.5 (0.06)	**0.005**	0.289	2 ± 0.5 (0.09)	0.317	0.125	0.514	0.073
	6W	2 ± 0.5 (0.07)			2 ± 0.5 (0.09)			0.294	0.117
Hurdle Step R	B	2 ± 0.5 (0.07)	**0.0001**	0.354	2 ± 0.5 (0.08)	1.000	0.000	0.113	0.177
	6W	3 ± 0.5 (0.06)			2 ± 0.5 (0.08)			**0.0001**	0.417
Hurdle Step L	B	3 ± 0.5 (0.07)	**0.002**	0.311	2 ± 0 (0.07)	0.096	0.208	**0.003**	0.333
	6W	3 ± 0.25 (0.06)			2 ± 0.5 (0.06)			**0.0001**	0.402
In-line Lunge R	B	2.5 ± 0.5(0.07)	**0.0001**	0.456	2 ± 0 (0.07)	1.000	0.000	**0.019**	0.262
	6W	3 ± 0 (0.04)			2 ± 0.25 (0.08)			**0.0001**	0.655
In-line Lunge L	B	2 ± 0.5 (0.07)	**0.001**	0.351	2 ± 0 (0.08)	0.096	0.208	**0.004**	0.319
	6W	3 ± 0 (0.05)			2 ± 0.5 (0.08)			**0.0001**	0.505
Shoulder Mobility R	B	3 ± 0 (0.05)	0.102	0.167	3 ± 0 (0.06)	0.317	0.125	0.896	0.015
	6W	3 ± 0 (0.02)			3 ± 0 (0.04)			0.340	0.107
Shoulder Mobility L	B	3 ± 0 (0.06)	**0.008**	0.270	3 ± 0 (0.06)	1.000	0.000	0.408	0.093
	6W	3 ± 0 (0.04)			3 ± 0 (0.06)			0.493	0.077
ASLR R	B	3 ± 0.5 (0.06)	**0.002**	0.311	2.5 ± 0.5 (0.10)	**0.005**	0.354	0.167	0.155
	6W	3 ± 0 (0.04)			3 ± 0.5 (0.08)			0.081	0.195
ASLR L	B	3 ± 0.5 (0.06)	**0.008**	0.273	3 ± 0.5 (0.10)	**0.020**	0.292	0.266	0.124
	6W	3 ± 0 (0.05)			3 ± 0.5 (0.08)			0.140	0.165
TSPU	B	2 ± 0.5 (0.11)	0.071	0.184	2 ± 1 (0.16)	0.083	0.217	0.181	0.150
	6W	3 ± 0.75 (0.12)			2 ± 1 (0.16)			0.118	0.175
Rotary stability R	B	2 ± 0 (0.05)	**0.0001**	0.357	2 ± 0 (0.0)	1.000	0.000	0.109	0.179
	6W	2 ± 0.5 (0.07)			2 ± 0 (0.0)			**0.0001**	0.453
Rotary stability L	B	2 ± 0 (0.05)	**0.005**	0.290	2 ± 0 (0.0)	1.000	0.000	0.055	0.251
	6W	2 ± 0.5 (0.07)			2 ± 0 (0.0)			**0.0001**	0.437
Composite score	B	17 ± 1.5 (0.23)	**0.0001**	0.522	16 ± 1.5 (0.31)	0.027	0.277	**0.019**	0.262
	6W	18 ± 1.5 (0.24)			16 ± 1.25 (0.31)			**0.0001**	0.486

ASLR—Active straight leg raise; TSPU—Trunk stability push-up; R—right side; L—left side; B—measurement at baseline; 6W—measurement after six weeks; *p* ^a^—*p* value between baseline and six week within each group; *p* ^b^—*p* value between study groups; ES ^a^—effect size (Cohen’s r) within each group; ES ^b^—effect size (Cohen’s r) between study groups; Cohen’s r effect size: <0.1—trivial; 0.1–0.3—small; 0.3–0.5—medium; >0.5–large; Values are expressed as Me ± IQR/2 (SEM)—Median ± half of interquartile range (Standard Error of Measurement); bold—statistically significant.

**Table 3:** Functional tests of major muscle groups at baseline and after six weeks of exercising.

Outcome Measure		Group 1 Mean ± SD (SEM)	*p* ^a^	ES ^a^	Group 2 Mean ± SD (SEM)	*p* ^a^	ES^a^	*p* ^b^	ES ^b^
Piriformis (external rotation muscles) [cm]	B	60.3 ± 0.4 (1.50)	**0.005**	0.391	60.1 ± 9.0 (1.60)	**0.001**	0.647	0.947	0.008
	6W	62.4 ± 10.3 (1.49)			58.0 ± 8.5 (1.51)			0.060	0.236
Iliopsoas L [cm]	B	9.3 ± 5.4 (0.78)	**0.0001**	0.608	6.5 ± 4.0 (0.70)	0.217	0.221	0.070	0.289
	6W	6.5 ± 4.8 (0.69)			7.0 ± 3.9 (0.70)			0.653	0.052
Iliopsoas R [cm]	B	8.9 ± 5.1 (0.73)	**0.0001**	0.486	6.2 ± 3.5 (0.62)	0.275	0.196	**0.007**	0.299
	6W	6.7 ± 4.7 (0.68)			6.7 ± 3.6 (0.64)			0.974	0.004
Tensor fasciae latae L [cm]	B	15.8 ± 7.0 (1.01)	**0.0001**	0.743	18.3 ± 9.1 (1.61)	0.149	0.257	0.202	0.172
	6W	20.8 ± 7.0 (1.02)			17.2 ± 6.5 (1.15)			0.061	0.272
Tensor fasciae latae R [cm	B	16.7 ± 6.8 (0.98)	**0.0001**	0.730	18.5 ± 9.0 (1.60)	0.488	0.125	0.338	0.131
	6W	22.2 ± 6.3 (0.91)			18.1 ± 6.7 (1.20)			0.080	0.327
Rectus femoris L [cm]	B	24.9 ± 4.0 (0.58)	**0.0001**	0.563	27.2 ± 3.4 (0.61)	0.290	0.190	**0.009**	0.299
	6W	23.5 ± 3.4 (0.50)			27.5 ± 3.2 (0.51)			**0.0001**	0.535
Rectus femoris R [cm]	B	24.7 ± 3.8 (0.54)	**0.0001**	0.519	27.1 ± 3.5 (0.59)	0.110	0.338	**0.005**	0.323
	6W	23.6 ± 3.4 (0.50)			27.6 ± 3.3 (0.60)			**0.0001**	0.525
Adductor muscles [cm]	B	65.1 ± 7.1 (1.03)	**0.0001**	0.488	72.2 ± 13.1 (2.32)	0.056	0.350	**0.008**	0.390
	6W	67.3 ± 7.1 (1.03)			70.5 ± 10.5 (1.85)			0.139	0.208
Quadratus lumborum L [cm]	B	21.8 ± 3.5 (0.50)	**0.002**	0.427	19.0 ± 3.5 (0.62)	0.909	0.021	**0.001**	0.384
	6W	23.1 ± 3.8 (0.54)			19.0 ± 3.2 (0.56)			**0.0001**	0.512
Quadratus lumborum R [cm]	B	22.8 ± 3.4 (0.49)	**0.003**	0.409	19.3 ± 3.6 (0.63)	1.000	0.000	**0.0001**	0.411
	6W	23.4 ± 3.1 (0.46)			19.3 ± 3.3 (0.59)			**0.0001**	0.559

R—right side; L—left side; cm—centimetres; B—measurement at baseline; 6W—measurement after six weeks; *p* ^a^—*p* value between baseline and six week within each group; *p* ^b^—*p* value between study groups; ES^a^—effect; size (Cohen’s r) within each group; ES^b^—effect size (Cohen’s r) between study groups; Cohen’s r effect size: <0.1—trivial; 0.1–0.3—small; 0.3–0.5—medium; >0.5—large; Values are expressed as Me ± IQR/2 (SEM)—Mean ± Standard Deviation (Standard Error of Measurement); bold—statistically significant.
